# AI-based disease category prediction model using symptoms from low-resource Ethiopian language: Afaan Oromo text

**DOI:** 10.1038/s41598-024-62278-7

**Published:** 2024-05-16

**Authors:** Etana Fikadu Dinsa, Mrinal Das, Teklu Urgessa Abebe

**Affiliations:** 1 Department of Computer Science and Engineering, Engineering and Technology, Wollega University, Oromia, Ethiopia; 2 Department of Data Science, Indian Institute of Technology Palakkad (IIT Palakkad), Palakkad, India; 3 Department of Computer Science and Engineering, Adama Science and Technology University, Adama, Ethiopia

**Keywords:** Artificial intelligence, Classification, Deep learning, Health data, Machine learning, Computer science, Information technology

## Abstract

Automated disease diagnosis and prediction, powered by AI, play a crucial role in enabling medical professionals to deliver effective care to patients. While such predictive tools have been extensively explored in resource-rich languages like English, this manuscript focuses on predicting disease categories automatically from symptoms documented in the Afaan Oromo language, employing various classification algorithms. This study encompasses machine learning techniques such as support vector machines, random forests, logistic regression, and Naïve Bayes, as well as deep learning approaches including LSTM, GRU, and Bi-LSTM. Due to the unavailability of a standard corpus, we prepared three data sets with different numbers of patient symptoms arranged into 10 categories. The two feature representations, TF-IDF and word embedding, were employed. The performance of the proposed methodology has been evaluated using accuracy, recall, precision, and F1 score. The experimental results show that, among machine learning models, the SVM model using TF-IDF had the highest accuracy and F1 score of 94.7%, while the LSTM model using word2vec embedding showed an accuracy rate of 95.7% and F1 score of 96.0% from deep learning models. To enhance the optimal performance of each model, several hyper-parameter tuning settings were used. This study shows that the LSTM model verifies to be the best of all the other models over the entire dataset.

## Introduction

In the field of healthcare industry, the study of disease identification plays a crucial role. Any cause or circumstance that leads to illness, pain, dysfunction, or eventually human death is called a disease^1^. Diseases can have an impact on a person’s mental and physical health, and they significantly manipulate the living styles of the affected person. The instrumental study of disease is called the pathological process. Clinical experts interpret the signs and symptoms that cause a disease^2–4^. Diagnosis has been well defined as the technique of identifying the disease from its indications and symptoms to determine its pathology. Another definition is that the steps of identifying a disease based on the individual’s signs and symptoms are called diagnosis^1,5^. Disease symptoms and their impacts on quality of life are crucial information for medical professionals, and their ability to identify them can help shape patient care and the drug development process^6–8^. An appropriate decision support system is needed to obtain correct diagnosis results with less time and expense. Classification of diseases based on several parameters is a complex task for health experts, but artificial intelligence would aid in detecting and handling such cases. Currently, the medical industry uses different artificial intelligence (AI) technologies to effectively diagnose illnesses. AI is a fundamental part of computer science, through which computer technologies become more intelligent. Learning is the most important thing for any intelligent system. Artificial intelligence makes the system more sensitive and activates the system to think^9^.

There are numerous methods in AI that are centered on learning, like deep learning, machine learning (ML), and data mining algorithms for medicine, which have accelerated in growth, focusing on the health of patients and their ability to predict diseases^2,10^. Some benefits of medical data analysis are: (a) patient-centered and structured information; (b) the ability to bunch the population into groups according to features such as diagnosis or disease symptoms; (c) the ability to carry out analyses of drug effectiveness and effects in people; and (d) clinical patterns^4^. Novel information technologies and computational methods can be used to improve the analysis and processing of medical data. The important task in data processing and analysis is text classification and clustering, which is a field of research that has gained thrust in the last few years^11^. These approaches are helpful for health data analysis since several medical datasets in the health industry, such as those on disease characterization, could be analyzed through different approaches to predictive analytics^12–15^.

This paper proposes a model that automatically predicts the disease category based on symptoms documented in the Afaan Oromo language using classification algorithms. This would give the physician a general idea of the user’s willingness to visit and reduce the time taken to determine the patient’s disease from handwritten materials. The output of this work can be used to automate manual systems for finding disease types by experts, reduce errors, and save human resources and time. We used natural language processing techniques^16^, which are cost-effective and have been demonstrated to be the right approaches for obtaining structured information^17,18^. The main objective of our study is to apply NLP techniques to the symptoms given by the user and then utilize ML and DL models to predict disease class labels. Finally, the prediction accuracy of the models was evaluated to determine which model provided the best performance.

The contributions of our research are:

- We developed an Afaan Oromo patient symptoms (AOPS) corpus that contains health text documents labeled in ten categories.
- We have developed word embedding (word2vec) from our corpus.
- We have conducted experiments with ML such as SVM (support vector machine), random forest, logistic regression, and Naïve Bayes and deep learning algorithms' such as LSTM (long short term memory), GRU (gated recurrent unit), and Bi-LSTM (bi-directional long short term memory).
- We compared the deep learning model’s performance with machine learning models and found that the DL model outperformed.
- From all the trained models, LSTM plus trained word2vec shows the best performance of all the other models by giving 95.7% accuracy and 96.0% F1 score.

The rest of the paper is structured as follows: Sect. "Background of Afaan Oromo Languages" presents the background of Afaan Oromo Languages; Sect. "Related work" discusses related work; Sect. "Back ground of relevant artificial intelligence methods" presents all about relevant artificial intelligence in the study; Sect. "Materials and methods" discusses materials and proposed methodology; Sect. "Experimental result and performance comparison" discusses all about the results; Sect. "Discussion" presents discussion; and finally, conclusion and future work are discussed in Sect. "Conclusion and future work".

## Background of Afaan Oromo languages

Afaan Oromo is a member of the Cushitic branch of the native Afro-Asiatic language spoken in many parts of Ethiopia and neighboring countries like Kenya, Djibouti, Tanzania, and Somalia, which have Horn of Africa coverage^19,20^. The biggest Cushitic language on the African continent is Hausa, followed by the Afaan Oromo languages^21,22^. Afaan Oromo is used by the majority ethnic group in Ethiopia, the Oromo people, which amounts to half of the total population of the country. It is also the working language of the Oromia regional state, which is the largest regional state in Ethiopia. Afaan Oromo is commonly used as a ‘written’ and ‘spoken’ language in the countries. With concern for the writing system of Afaan Oromo, “Qubee” (a ‘Latin-based alphabet’) has been implemented and has become the official script of Afaan Oromo starting in 1991^21,23^. Since then, it has been a written language, school language, public social media, social issues, religious party, political affairs, technology, and a working language^19^. Afaan Oromo and English have different sentence structures. Afaan Oromo uses subject-object-verb order (SOV) language. English uses subject-verb-object (SVO). This is the main reason that the model developed for English is not functional for Afaan Oromo.

## Related work

This section focuses on several automated methods applied to health data and classification problems using various techniques. In addition to this, we review some related topics with different domains and the same methods as the current study.

### Afaan Oromo related researches

In the work^24^, which focuses on variations of profound DL models such as convolutional neural networks (CNN), LSTMs, Bi-LSTMs, LSTM, GRU, and CNN-LSTM are examined to evaluate their viability in identifying Afaan Oromo hate speeches. They prepared the Afaan Oromo Corpus for hate speech detection. Considering the dataset size examined in their paper, the resultant performance of the deep learning model at identifying Afaan Oromo hate speech is promising. Their finding shows that the best performance was showcased by the Bi-LSTM, with a weighted classification F1 score of 91%. In paper^25^, the Afaan Oromo emotion detection model is developed using feed-forward neural networks, LSTM, and Bi-LSTM algorithms. The purpose of their work is to categorize sentences into emotion classes. They compared these algorithms and found that Bi-LSTM would achieve better performance. They achieved an accuracy of 66%, 78%, and 83% using Feed Forward Neural Network, LSTM, and Bi-LSTM, respectively. Based on experimental results, they concluded that growing the dataset size, tuning hyper-parameters properly, and trying different algorithms can enhance the performance of the model. Research done by^26^ was sentence-level sentiment analysis for multiple classes in Ethiopian language, Afaan Oromo text, to analyze the performances of selected supervised machine learning approaches ( SVM and RF). From their experiment, the result shows SVM performed with accuracy 90% and RF achieved an accuracy of 89% using the collected Afaan Oromo Twitter dataset with 1810 corpus size. They also criticize the impact of the unavailability of the standard dataset of Afaan Oromo text on their study.

### Research done on english text document

The work^27^ has experimented with both ML and DL methods on patient symptoms. In their work, they used a small dataset in the Bengali language from DL family CNN performed best with 82.27% accuracy when compared to ML classifiers. When they increased the number of documents in the dataset, they achieved CNN accuracy of 94.1% and RF accuracy of 94.6%, which is superior.Finally, they manually tested the system with RF classifier because RF gives the highest accuracy and the system predicts the specialist that matches the actual class label. They recommend that expanding the dataset will help to improve the system’s accuracy with more disease-specific specialist predictions. In the study^28^ ML Approach to Classifying Self-Reported Health Status was studied. They suggest using a selected machine learning algorithm to classify patient-reported outcomes using activity tracker patient’s data with stable ischemic heart disease. The study shows that activity trackers can be used to categorize patient health status over time using a hidden Markov model. This technique could play a future role in remotely monitoring a patient's health status in a clinically significant manner.

The work^18^ identified patient-reported symptoms and the impact on quality of life by categorizing unstructured, qualitative written data from interviews with cancer patients using unique natural language processing (NLP) techniques. From patient interviews, multiclass texts were accurately classified by NLP models. In their experiment at the paragraph and sentence levels, the BERT model consistently beats all the other models. In the study^7^, they evaluate patient sentiment on the quality of service provided by healthcare and classify it as high or low by analyzing text and photographic contents on physician rating websites using baseline machine learning and the CNN-LSTM algorithm. This study used the improved computational techniques by merging novel textual and visual features. In their work, deep learning models provide better predictive performance when compared to baseline ML models. The research done by^29^ hypothesizes that NLP techniques can aid in understanding patients’ communication about headache disorders. The study indicates that machine learning algorithms have the potential to classify patient self-reported narratives of migraines or cluster headaches with good performance. NLP shows its capability to differentiate relevant linguistic aspects in narratives from patients with diverse headache disorders and determines relevance in clinical information extraction. The potential benefits on the classification performance of larger datasets and neural NLP methods can be recommended for future work by the author.

The paper^30^ proposes a deep learning-based approach for textual document classification. In their experimental result, LSTM remembers the order of presented text data, and it performs with 92% accuracy over the Titanic dataset. LSTM’s have the property of removing unnecessary information’s and being able to remember the sequence of the text, which makes them an excellent tool for text classification compared to ML techniques. In the work^31^ selected machine learning classifiers and deep learning classifiers are implemented using word embedding features for the purpose of hotel review classification. The study reveals the deep neural network (DNN) architecture, which provides 97% accuracy to predict the review class. LSTM sequence modeling and word embedding help the model to train well and yield better results in their work. The results showed that their proposed hybrid model outperforms multi-layer perceptron (MLP), CNN, and LSTM models in terms of scored accuracy, recall, and F1_scores. The work^32^ studies the application of DL in text categorization. They combined it with textual characteristics and used the double bi-directional gated recurrent unit (GRU) + attention DL model to predict news hotspots, and they reached good results. The summery of this related literature are presented in Table 1.

**Table 1:** Summary of related papers.

Reference	Approaches	Number of classes	Dataset domain
27	ML and DL	9 classes	Health text in Bengali language
28	ML	7 classes	Health text in English
18	NLP models	3 classes	Health text in English
7	DL	2 classes	Opinion on healthcare service
29	NLP and ML	2 classes	Clinical text in English
30	DL	2 classes	Titanic dataset in english
31	DL	2 classes	Customer review in English
24	DL	4 classes	Afaan Oromo Hate speech dataset
32	DL	2 classes	English news classification
26	ML	5 classes	Sentiment analysis from Afaan Oromo twitter dataset
25	DL	5 classes	Afaan Oromo emotion detection dataset
Current work	ML and DL	10 classes	Afaan Oromo health text data

From this literature study, the researcher has come to the conclusion that there is no disease category prediction model for Afaan Oromo health text documents available, although there are some health text classification, prediction, and chat-bots available in English and other languages. One of the main reasons behind this scarcity is that, according to this literature study, there is no organized Afaan Oromo dataset available that can be used to diagnose diseases and classify them into specific groups. Based on this, determining which ML and DL algorithms performed the best for disease category prediction from symptoms in Afaan Oromo is the primary focus of the current study.

## Back ground of relevant artificial intelligence methods

Artificial intelligence (AI) tools will supplement and enhance human labor, not take its place, in the case of physicians and other healthcare professionals. AI is ready to assist medical staff in a variety of duties, including patient outreach, clinical recording, disease diagnosis and prediction, administrative workflow, and specialist support like medical device automation, image analysis, and patient information monitoring and assistance^33^. To implement such AI tasks in healthcare, the use of machine learning and deep learning algorithms plays an important role.

In this study, the researcher employs machine learning such as SVM (support vector machine), random forest, logistic regression, Naïve Bayes, and deep learning algorithms' such as LSTM (long short term memory), GRU (gated recurrent unit), and Bi-LSTM (bi-directional long short memory) for the experiment and development of the prediction model.

### Traditional machine learning algorithms

#### Random forest (RF)

The RF Classifier is a supervised ML technique that can resolve both regression and classification problems. It is based on an ensemble of decision trees and the application of the bagging scheme to produce the necessary prediction for class label^34^. The training data is fitted into the RF classification models, and the performance dataset has been evaluated. RF has many advantages, which makes it a noble algorithm for classification problems. With the default hyper-parameters, a good prediction result can be achieved. It also reduces the risk of over-fitting the model, as an RF classifier is built on multiple decision trees, and the output is based on the majority voting or averaging. The RF classifier mathematically works as given by the formulas in Eqs. 1 and 2.

Let: T is set of decision tree, X is given data point, Y is final prediction, and C is set all predicted class labels ci obtained from decision tree.

For each *ti* decision tree in T and ci is predicted class label by ith tree for input X

1 $${\text{ci}} = {\text{predict }}\left( {ti,{\text{X}}} \right)$$

2 $${\text{Y}} = {\text{mode}}\left( {\text{C}} \right){ }$$

#### Support vector classifier (SVC)

**Figure 1:**
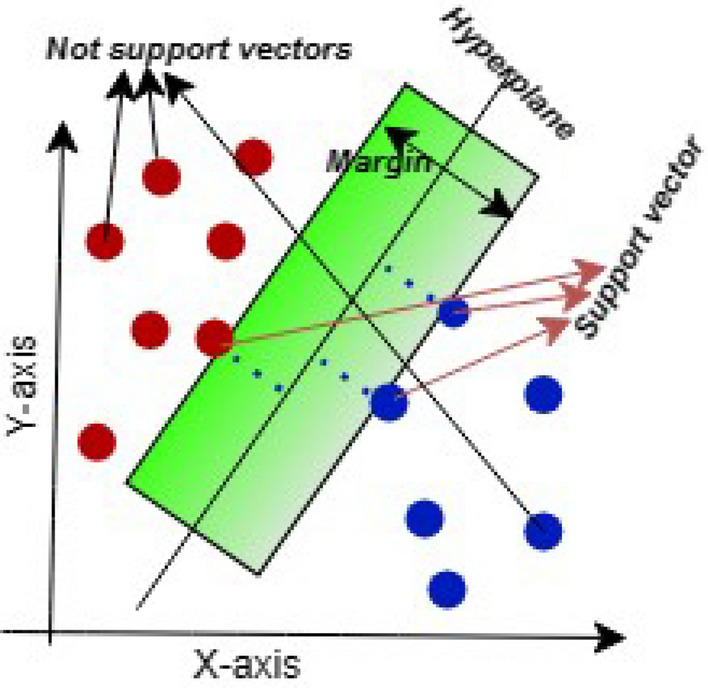
The block diagram of SVM classifier.

One of the supervised learning techniques in ML that can be used in classification problems is known as SVM^26^. It is an approach in ML that can aid in classifying large amounts of data. The SVM classifier separates data points using a hyper-plane in multidimensional space to separate them into different classes. Its main target is to find the maximum marginal hyper-plane between the support vectors that divide the dataset into classes in the best possible way, as shown in Fig. 1. In our study, we have used the ‘rbf’ kernel and gamma value (0.001) to determine the shapes of the decision boundary. Then we fit the training data into the SVM model and analyzed its performance.

#### Logistic Regression (LR)

Classifier LR is another ML algorithm that can be used for binary classification or multi-class classification^35,36^. It is widely used as it is easy to implement. It uses the “one-vs-rest” (OvR) strategy in multi-class classification. The general formula for LR is given in Eq. 3. In our experiment, logistic regression has been applied to evaluate the performance over a given dataset.

3 $$p\left( {y = x/k} \right) = \frac{1}{{1 + e^{ - ax} }}$$

where: *p*(*y* = *x*/*k*) is the probability of input *k* belongs to *x* class, *ax* is the combination of input features weighted by the *θ*_*x*_ parameters, *ax* = *θ*_*x0*_ + *θ*_*x1k1*_ + *θ*_*x2k2*_ + *…* + *θ*_*xnkn*_ and *e* is the base of natural logarithm (about equal to 2.71828).

#### Naïve Bayes

A multinomial Naïve Bayes (NBMN) classifier model is a specific instance of a Naive Bayes classifier that is designed to determine term frequency, i.e. the number of times a term occurs in a document^37^. Considering the fact that a term may be pivotal in deciding the concepts of the document, this property of this model makes it a covered choice for document classification. Also, term frequency is helpful in deciding whether the term is useful in our analysis or not. Naive Bayes uses the Bayes rules as described in Eq. 4.

4 $$p\left( {h/D} \right) = \frac{{p\left( {D/h} \right)p\left( h \right)}}{p\left( D \right)}$$

where: *P*(*h*) is the probability of *h* occurring, *P*(*D*) is the probability of *D* occurring, *P*(*h*|*D*) is the probability of *h* occurring given evidence *D* has already occurred, and *P*(*D*|*h*) is the probability of *D* occurring given evidence h has already occurred.

### Deep learning models

#### Long short term memory (LSTM)

**Figure 2:**
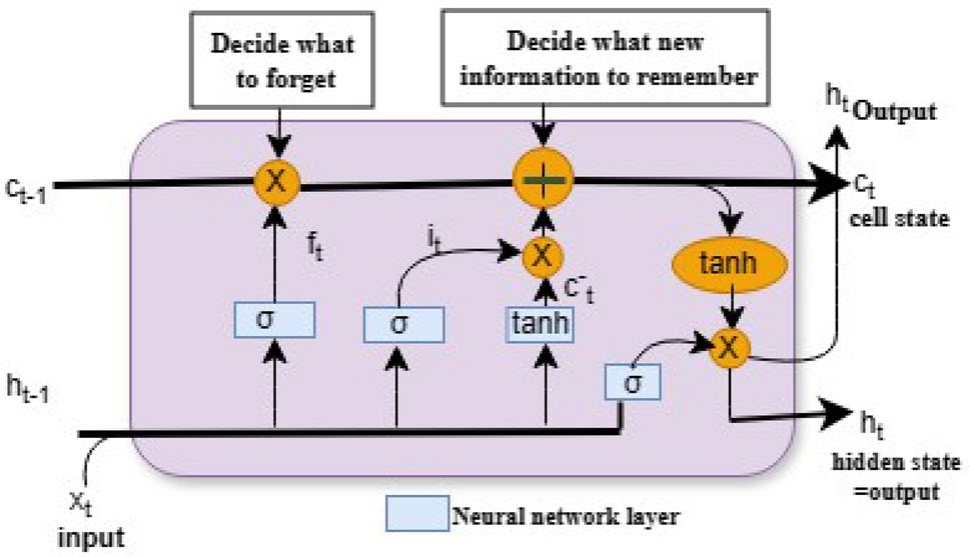
The LSTM models architecture.

The LSTM model is a deep neural network, specifically an extension of a recurrent neural network. It is an improved RNN architecture that uses three gating mechanisms: input, forget, and output gates that regulate data flow^38^. These gates are used to determine which data in the prior state should be taken or forgotten in the current state^39^. The forget gate helps decide whether information can go between network tiers. The input gate determines the relevance of information and aids the forget function in removing irrelevant data and allowing other layers to learn the data needed for prediction. The output gate is the LSTM network’s last gate, which helps in determining the network’s next hidden state, in which information passes via the activation function. In Fig. 2, the internal architecture of the LSTM model is described clearly. In our study, we used spatial dropout and dropout layers to avoid over-fitting issues. The detailed LSTM parameters used for this experiment are discussed in the proposed methodology section.

#### Gated recurrent unit (GRU)

GRU is another extension of the RNN that is similar to LSTMs and addresses the problems of short-term memory in RNNs. However, GRUs have two gates instead of three and do not include the cell state^40^. Therefore, GRUs is structurally simpler than LSTM and train faster due to fewer tensor operations. However, this does not mean that they are superior to LSTMs. Which one is better is depends on the use case^38^. In this work, we used GRU with a unit value of 64, a dropout value of 0.2, and an epoch 10 when we trained our model.

#### Bi-directional LSTM

**Figure 3:**
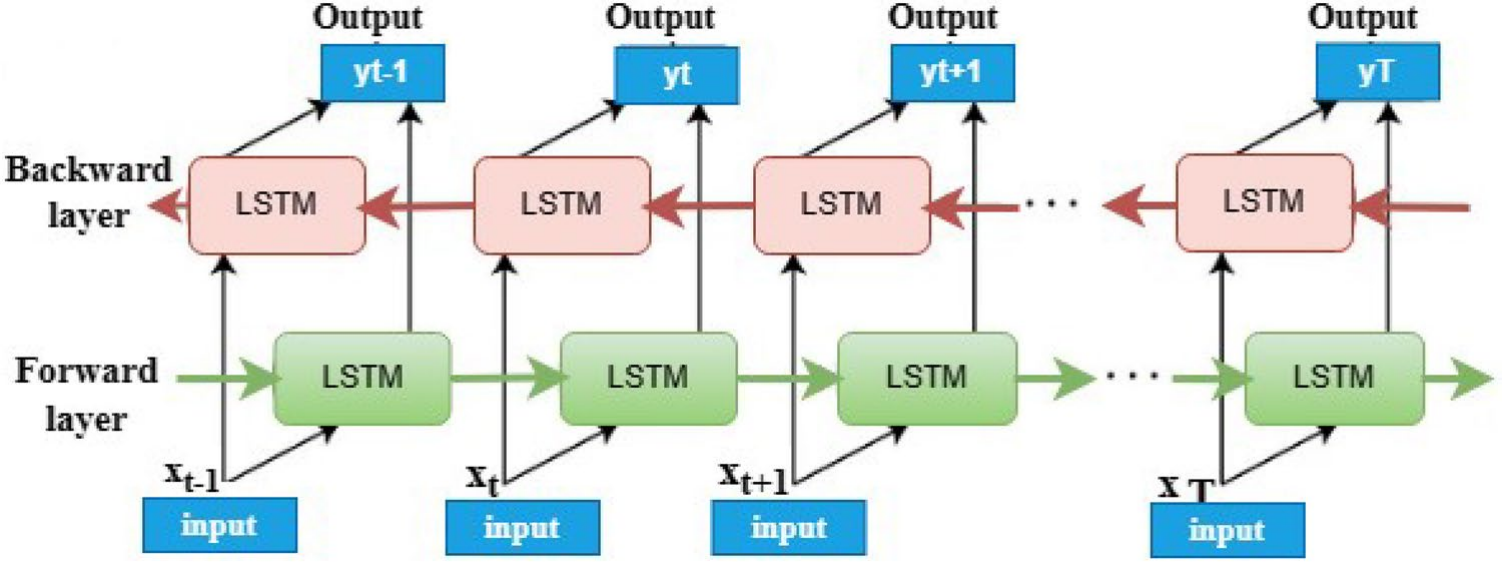
The Bi-LSTM architectural diagram.

Bidirectional LSTMs^38^ are an extension of LSTM that can increase model performances on sequences of classification problems. Bi-LSTMs are used for problems where all data is time-stamped of the input sequence. In that situation, bidirectional LSTMs trained two instead of one LSTM on the input sequence to provide the final results. Bi-LSTM reverses the direction of the flow of information by adding one more LSTM layer, as presented in Fig. 3. In this work, we used bidirectional LSTMs for our implementation.

#### BERT and GPT

Both Bidirectional Encoder Representations from Transformers and Generative Pre-trained Transformer are deep learning transformer models that are pre-trained on large corpora of English^41–43^. Being pre-trained with English text, those models are not useful for anything other than English. To train these models for other languages using the same architecture, it is necessary to have a large corpus of similar size. In the current paper, we do not include those models because we are restricted by limited datasets available in Afaan Oromo as of now, which is not sufficient to train them.

## Materials and methods

In this section, we describe an overview of the data used for the experiment, data processing techniques, word representation techniques, and some selected algorithms from ML and DL used in our work.

### Data collection

**Figure 4:**
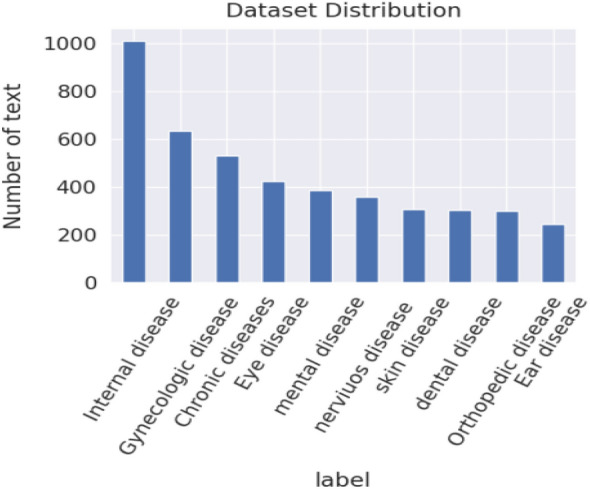
Representation of the total document per each class of our dataset-AOPS3.

The lack of comprehensive evaluation on openly accessible datasets for the Afaan Oromo language is a crucial drawback of the health-related text classification technique. Current studies are based on gathered datasets. We collected disease-related documents from various healthcare industries and available online resources to train and test the proposed model. Since there is no publicly available Afaan Oromo health-related text document corpus, we prepared a corpus of Afaan Oromo patient symptoms (AOPS) data in the form of a comma-separated file (CSV) with the corresponding categories. In this paper, disease symptoms are the same as patient symptoms, and they are interchangeably used. The data that was collected simply contains symptoms of the disease; no personal information about any individuals has been included. We used three experts to annotate the collected data. They are Afaan Oromo native speakers, and they are domain experts. Some of the symptoms we gathered are normally associated with clear descriptions and classes. To confirm whether they are correctly assigned, and for those which have not been assigned, we use these experts. They work on which symptoms should be assigned to which class label and which keywords correspond to each class label to annotate the data. Each symptom is identified by its own keywords. The more similar symptoms are classified under the same class label. We manage the inter-annotator agreements among annotators by majority. The overviews of the datasets we used in the study are shown in Table 2, and the class distribution is presented in Fig. 4. Table 3 presents the sample record from our AOPS dataset. The first column gives the problem statement in the Afaan Oromo language; the second column gives its meaning in English; and the third column contains the corresponding class label.

**Table 2:** Number of documents in Afaan Oromo patient symptoms (AOPS): AOPS1, AOPS2, and AOPS3.

Dataset	Total data	Number of class
AOPS1	1500 symptoms document	10
AOPS2	3000 symptoms document	10
AOPS3	4500 symptoms document	10

**Table 3:** Sample records received from AOPS show some symptoms with their meanings in English and class labels.

Symptoms in Afaan Oromo language	Symptoms meaning in English	Category
Naannoo kalee dhukkubbiin qaba	I have pain around kidneys	Internal disease
Dugda kootti dhukkubbiin natti dhagahama, socho'uu hin danda'u	I feel pain in my back, I can't move	Nervous disease
Dhiphina sammuu qaba	I have depression	Mental disease
Halluu adda addaan gogaa koo irraatti arge	I saw different colors on my skin	Skin disease

### Data preprocessing

**Figure 5:**
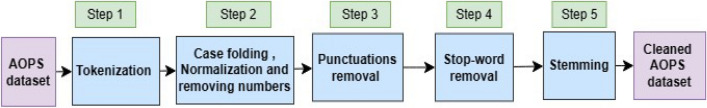
AOPS data preprocessing steps.

Preprocessing is an essential step before initiating the classification process^44^. Successful preprocessing actions affect the classification result^31^. The Afaan Oromo patient disease symptoms we gathered contain noisy, informal language, including unnecessary punctuation, the use of non-standard abbreviations, and capitalization. The collected data has many punctuation marks, capital letters, special characters, stop words, and numerical values. These are useless for the method of completing the classification process. Our dataset must be preprocessed before beginning the classification process to improve the performance of the model. The necessary AOPS preprocessing steps in this work are illustrated in Fig. 5. For instance, the description of the AOPS dataset after and before preprocessing is shown in Table 4.

**Table 4:** Sample description of the AOPS used for experiments before and after pre-processing.

AOPS before preprocessing	AOPS after preprocessing
Garaacha koo irraa na guba naa deeffachisa nyaata na dhowwa	garaach gub deeffachis nyaat dhoww
dugda na kutaa, natti bulluqa gadi hin jedhu oli hin jedhu kanaan baay'een rakkadha	dugda kuta bulluq gadi oli jedhu kanaan baay'ee rakkadh
IJATU na dhukkuba, keessa na waraana	ija dhukkub keess waraan
ulfatu irra ba'ee dhiigatu dhaabbachu dide	ulfa irra ba'e dhiig dhaabbach dide

#### Tokenization

Is the process of breaking a text into n-grams. Tokens can be separated by white space characters in Afaan Oromo sentences.

#### Punctuation removal

Punctuations are no actual importance when it comes to the analysis of the data. So, the better practice of data analysis comprises the removal of punctuations beforehand.

#### Normalization

In the Afaan Oromo writing system, there are different contraction words, and there is no written rule requiring us to use them in Afaan Oromo words. As a result, there is inconsistency in the writing of terms. So, before going into the training model, we have to normalize them. Example: BFO: “Biiroo fayyaa Oromiyaa”.

#### Remove stop-words

Stop-words are a group of irrelevant, most frequently occurring words that are not important for further classification. They have a much smaller purpose and have less grammatical constraints^45^. Consequently, we must eliminate them to reduce the low-level information in the text by only focusing on the vital information. However, there is no standard stop-word list for the Afaan Oromo language. To exclude them, we had to develop new stop-word lists for Afaan Oromo. For instance: ‘hin’, ‘wan’, ‘fi’, etc.

#### Stemming

This is the process of replacing the word with its root or stem. The advantage of stemming is that it simplifies word contrasts, as we don't have to deal with challenging grammatical changes to the word^46^. Due to the complexity of the morphological structure and the lack of developed stems for the languages, so, stemming Afaan Oromo texts faces several difficulties. Postfixes of each word in the Afaan Oromo text document corpus were identified. The length of postfixes to be removed from root words was decided by language expert. For example, the words “deeme”, “deemte”, “deeman”, and “deemaniiru” all could be stemmed to the word “deem”^47^. The researcher used python programming to implement stemming that will remove various suffixes, reduce the number of words, have exactly matching stems, and save memory space and time.

### Feature representations

Document classification involves the transformation of documents into feature vectors^48^. In our study, we used word2vec for the deep learning model and TF-IDF for the machine learning model. Word embedding is foundational to NLP and represents the words in a text in an R-dimensional vector space, thereby enabling the capture of semantics, semantic similarity between words, and syntactic information for words.

A. Term Frequency-Inverse Document Frequency.

To determine the significance of terms in a classification, the TF-IDF method is used since terms are not enough to differentiate one document from the others. This approach is based on the TF-IDF score of every term in the document, not relying on frequency. The algorithm works similarly to the bag of words, but the word count is replaced with the TF-IDF score of each term. The TF-IDF score of a given term t in a document will be formulated as Eq. 6.

6 $${\text{TF * IDF}}\left( {{\text{t}},{\text{d}}} \right) = {\text{TF}}\left( {t,d} \right){\text{ * log}}^{N/DFt}$$

where *N* denotes the total number of documents in the document, DF denotes document frequency, t denotes the term, and d denotes the document.

B. word2vec.

Word embedding via Word2vec was proposed by Mikolov^49^. Word2vec is a model that helps us represent the distributed representation of words in a corpus. In general, word2vec is an algorithm that accepts text as input and returns vectored representations of that text as output. Word2vec begins with a collection of randomly selected vector terms that scan the data set in a logical order while maintaining a background window around each term and its neighbors. The target word and its context are used by word2vec to decide how they act when they traverse the corpus. However, there is no pre-trained word-to-vector model for Afaan Oromo; we trained the model from scratch by using the collected corpus. Deep learning models are better at word2vec than other feature extraction.

### Proposed methodology

**Figure 6:**
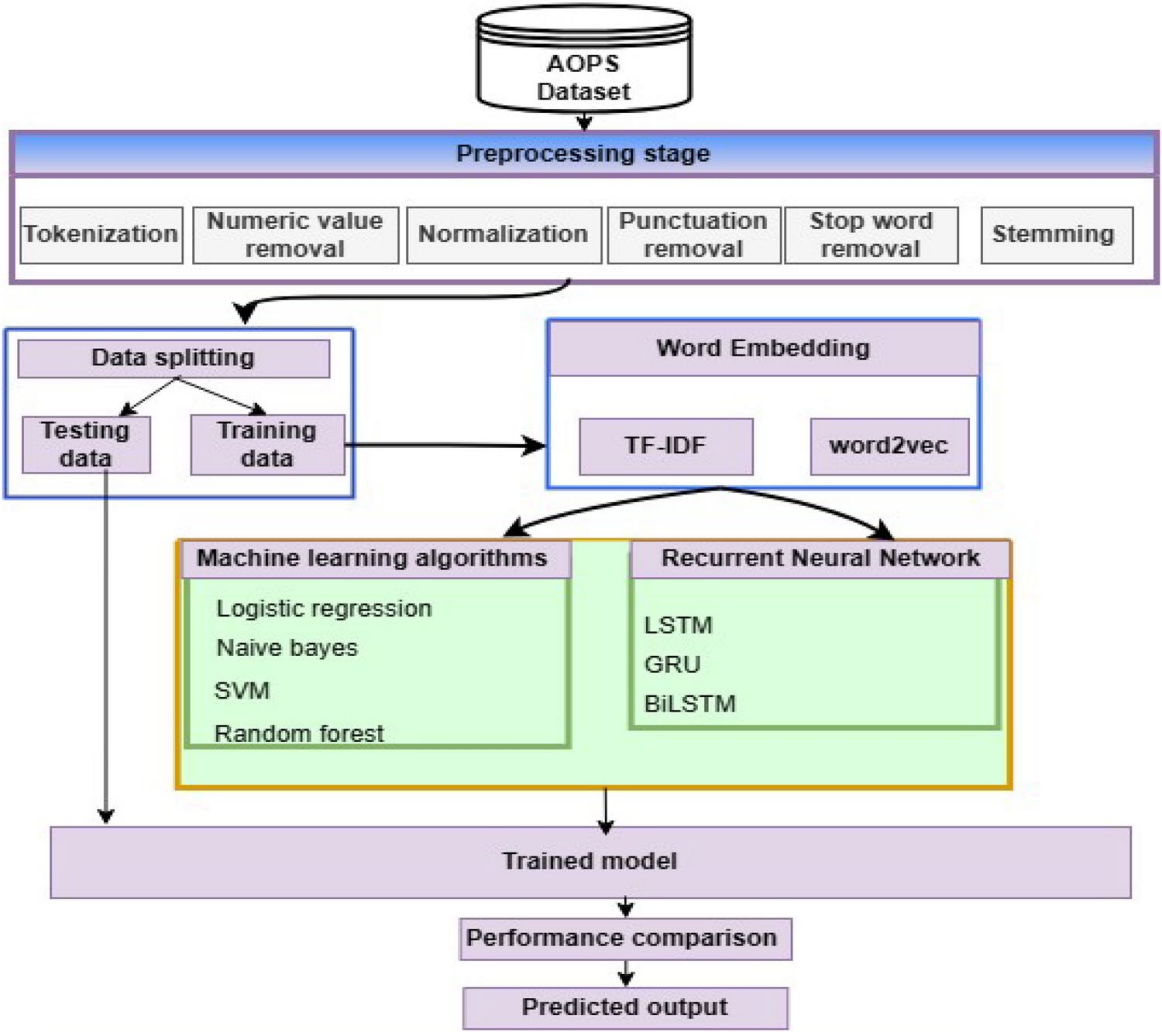
The architectural diagram of the overall proposed methodology.

This section presents the proposed framework used in this study. The aim of the paper is to predict and classify different disease classes using patient symptoms data gathered in Afaan Oromo text form. AI models can potentially optimize the classification accuracy of traditional classifiers. For this reason, our study aims to utilize both machine learning and deep learning approaches for health data disease category prediction. The architectural diagram of the proposed methodology for our study is given in Fig. 6. First, we start by collecting data from the healthcare industry available around patient symptoms written in Afaan Oromo. Then, we pooled them into one corpus divided into ten categories. The text preprocessing takes place by removing punctuation and numeric values. The texts go through tokenization to eradicate Afaan Oromo stop-words and stem the words. We divide the data set by 80% for training and 20% for testing ratio. We used ML classifiers, including SVM, LR, RF, and NB, as the baseline models and trained by extracting the features of the cleaned symptoms texts using the TF-IDF techniques. Then it was determined whether other DL classifiers, including LSTM, GRU, and Bi-LSTM could improve the accuracy of the ML models. In our study, we used LSTM layers with 100 neurons, and the activation function “soft-max” significantly outperformed other methods for identifying complicated aspects of the text. Soft-max is an activation function used in our experiment for the purpose of solving multi-class classification problems by providing a probability distribution over multiple classes. We used a word length of 200 on average. For sentences with fewer than 200 words, the index is filled by appending a zero at the end until it reaches 199 indexes. The embedding dimension used in this experiment is 100, which describes the size of the vector representation of words. The network parameters were regularized with a dropout rate of 0.2. We used this dropout to help increase the generalization capacity of the training model by decreasing the possibility of over-fitting. Categorical cross-entropy was utilized as a loss function, which is well suited to optimizing model performance. The training models are noble at 10 epochs and batch size 64. Adam is an optimization algorithm that is used to update the parameters (biases and weights) of the training models to minimize the loss function. Adam used in this implementation is the default learning rate, which is 0.001. To prevent over-fitting in the training, the callback function is incorporated with a patience of 5 epochs and a minimum delta of 0.0001. Table 5 shows the LSTM hyper-parameters used in our study. Finally, in the proposed methodology for the ML and DL models used, we have done performance evaluation and comparison in terms of accuracy, recall, F-measure, and precision^50^.

**Table 5:** The LSTM model hyper parameters used in this study.

Hyper parameter	Value
Embedding dimension	100
LSTM layer	100 hidden units
Batch size	64
Dropout	0.2
Activation	Softmax
Optimization	Adam
Loss function	Categorical-cross entropy

## Experimental result and performance comparison

**Figure 7:**
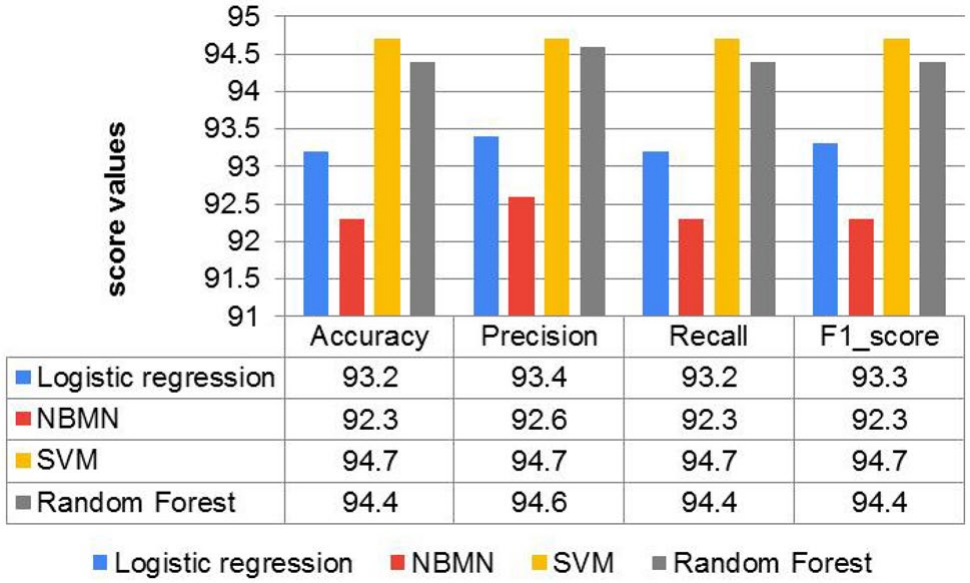
Performance comparisons of all the Machine learning models on AOPS3.

**Figure 8:**
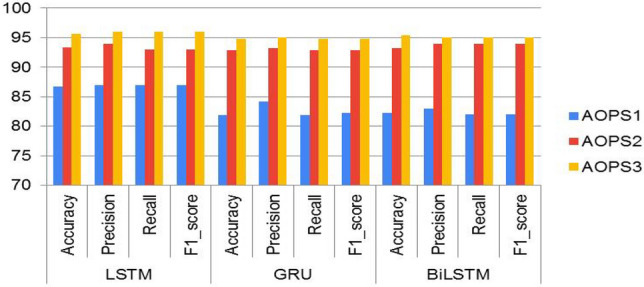
Performance comparisons of all the DL models on AOPS1, AOPS2 and AOPS3.

**Figure 9:**
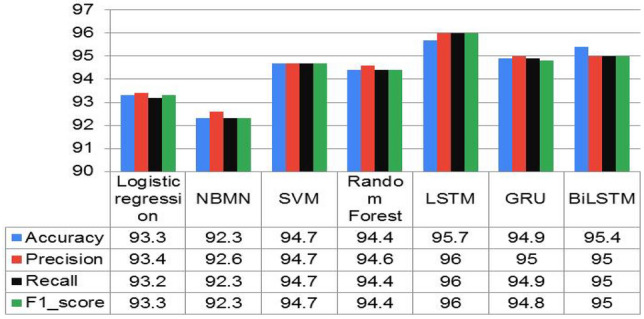
Performance comparison of the DL with ML models on AOPS3.

The performance of ML algorithms and DL classifiers applied to the entire dataset in this paper is presented in this section. Using evaluation matrices such as accuracy, recall, precision, and f1-score, the predicted labels of the test data were evaluated and compared to the actual labels to assess the model’s performance. Figure 7 shows the classification results for each baseline model using the TF-IDF techniques. Among ML models, the SVM model trained with the TF-IDF had the highest accuracy of 94.7% and the best F1 score of 94.7%. The RF model attained an accuracy of 94.4%, and the F1 score of 94.4% is the second outperforming model. With an accuracy of 92.3%, the Naïve Bayes model shows the least performance when baseline models are compared. Now we will compare the outcomes of deep learning models to see if they can outperform the results of baseline SVM models. The classification scores of each DL classifier trained on word embedding (trained word2vec) are illustrated in Fig. 8. In this paper, we used LSTM, Bi-LSTM, and GRU from deep learning algorithms to compare with machine learning algorithms, and they showed better performance, as presented in Table 6. From DL classier LSTM model outperforms the baseline SVM model by an accuracy rate of 95.7% and F1 score 96.0%, as illustrated in Fig. 9. The findings of this study are that the GRU, Bi-LSTM, and LSTM techniques demonstrate that deep learning algorithms utilizing the word2vec approach can outperform machine learning models.

**Table 6:** Performance of each ML with TF-IDF and DL model with word2vec on AOPS1.

Approach	Model	Accuracy	Precision	Recall	F1_Score
ML	Logistic Regression	78.9	82.7	78.9	79.2
	NBMN	75.2	79.2	75.2	74.6
	**SVM**	**83.7**	**85.8**	**83.7**	**84.1**
	Random Forest	80.0	82.3	80.0	80.3
DL	**LSTM**	**86.7**	**87.0**	**87.0**	**87.0**
	GRU	81.9	84.2	81.9	82.2
	BiLSTM	82.3	83.0	82.0	82.0

Significant values are in [bold].

### Result validation

In this work, we used the K-fold cross-validation method to validate all the baseline models because it is easy to implement. The results have a higher level of informative value than traditional validation methods. The approach includes just one parameter, K, which specifies the number of groups into which a given data sample should be divided. With K = 5, we would be able to get five metric results and have a deeper look into our model’s performance, and get a higher level of reliability. The K-fold cross-validation method was performed to cross-validate our machine-learning models with the TF-IDF feature extractor. We calculated the mean accuracy and variance for each fold to validate the performance of baseline ML models, as shown in Fig. 7. Lower variance indicates the learning algorithm’s limited sensitivity to the details of the training data, while higher mean accuracy exhibits the model’s ability to execute text classification accurately. The SVM model achieved the highest mean accuracy of 94.7% on fivefold cross-validation. The mean accuracy obtained by cross-validation is similar to the results of baseline models without cross-validation, as shown in Fig. 7. The researcher also applied trained Word2Vec feature extraction to selected ML, and the result is lower than that of the TF-IDF results. From the ML classifier, the highest accuracy, which is 92.3%, is recorded by Random Forest with Word2Vec on dataset AOPS3, which is presented in Table 6. The multi-nominal Naive Bayes algorithm is not applicable with trained word2vec techniques since the document will have certain negative values when we employ document and word vectors.

## Discussion

**Figure 10:**
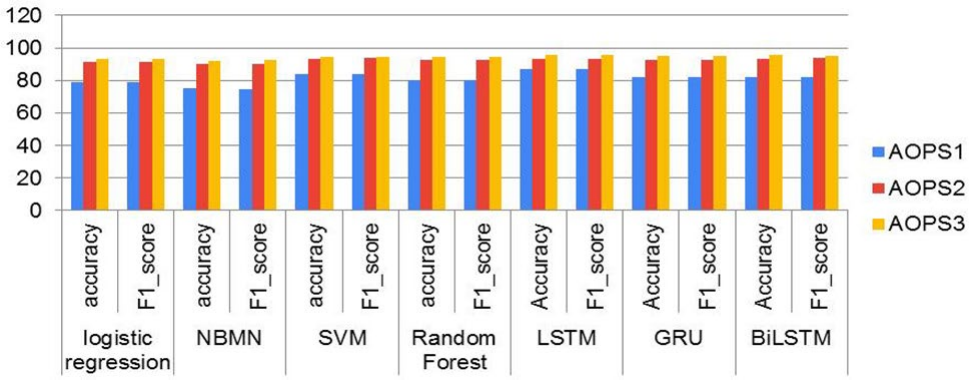
Result comparison of the LSTM model with other DL and ML algorithms on all AOPS1, AOPS2, and AOPS3.

**Figure 11:**
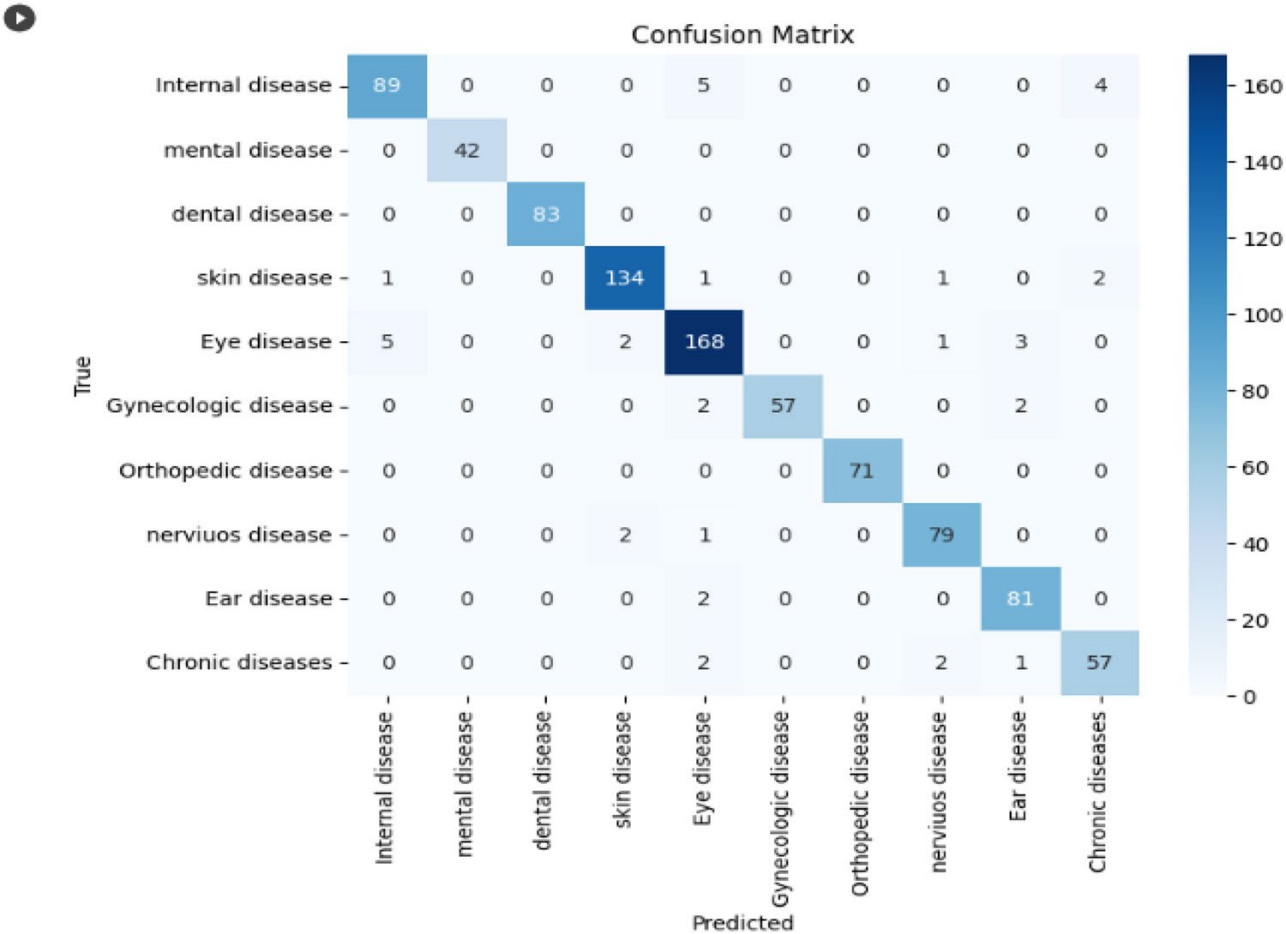
Confusion Matrix for LSTM model using Word2vec.

In this study, the proposed methodology was evaluated using performance evaluation metrics such as accuracy, precision, recall, and F1_score. Four ML algorithms, like logistic regression, SVM, Naïve Bayes, and Random Forest, and deep-learning algorithms like long-short-term memory, bidirectional LSTM, and Gated recurrent unit, were implemented in our study. Deep learning models have shown healthy results on all three datasets, as shown in Tables7, 8 and 9. To the extent of our knowledge, this is the first time to use this method to analyze and predict disease from Afaan Oromo health data based on symptoms. In this study, the LSTM model has shown superiority in performance as compared to GRU and BiLSTM over the entire dataset, as presented in Fig. 8. Over-fitting in the LSTM model is prevented by reducing the difficulties of the model and by applying regularization techniques in this work. To show the performance comparison of all models with the three datasets, we select accuracy and F1_scores as presented in Fig. 10. It is observed that the LSTM model is the best performer of all the models. From this, the researcher concludes that as the number of documents increases, the performance of LSTM approach improves well when compared with others. The complete LSTM model training and testing accuracy throughout the execution of all epochs over AOPS3 is shown in Fig. 11. However, it is also important to consider the limitations of this study. We used a limited number of datasets, and there are no standard accessible datasets in the case of selected languages and study domains. In the future, we aim to apply other advanced NLP models by collecting a larger number of datasets in the same domain.

**Table 7:** Performance of each ML with TF-IDF and DL model with word2vec on AOPS2.

Approach	Model	Accuracy	Precision	Recall	F1_Score
ML	Logistic Regression	91.1	91.5	91.1	91.1
	NBMN	90.4	90.8	90.4	90.3
	**SVM**	**93.2**	**93.9**	**93.7**	**93.8**
	Random Forest	92.8	92.9	92.8	92.8
DL	**LSTM**	**93.3**	**94.0**	**93.5**	**93.9**
	GRU	92.8	93.2	92.8	92.9
	BiLSTM	93.2	94.0	94.0	94.0

Significant values are in [bold].

**Table 8:** Performance of each ML with TF-IDF and DL model with word2vec on AOPS3.

Approach	Model	Accuracy	Precision	Recall	F1_score
ML	Logistic Regression	93.2	93.4	93.2	93.3
	NBMN	92.3	92.6	92.3	92.3
	**SVM**	**94.7**	**94.7**	**94.7**	**94.7**
	Random Forest	94.4	94.6	94.4	94.4
DL	**LSTM**	**95.7**	**96.0**	**96.0**	**96.0**
	GRU	94.9	95.0	94.9	94.8
	BiLSTM	95.4	95.0	95.0	95.0

Significant values are in [bold].

**Table 9:** Presents the accuracy of each machine learning models with trained word2vec and TF-IDF on dataset AOPS3.

ML model	Word2vec	TF-IDF
Logistic Regression	68.7	93.2
Random Forest	92.3	94.4
SVM	84.0	94.7
NBMN	-	92.3

**Figure 12:**
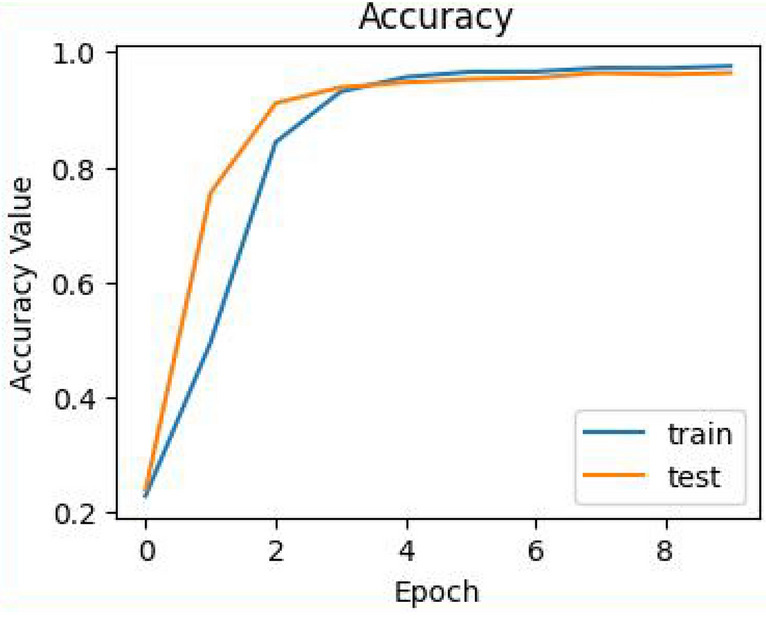
Training and testing accuracy of LSTM model with Word2vec.

Figure 12 describes the LSTM methods of confusion matrix that can measure the validity of a classification task. The confusion matrix is used to analyze the distribution and overlapping of successfully and erroneously predicted labels concerning other labels. In this disease category prediction model, each class was examined with the color intensity from this confusion matrix. The model predicts that mental and dental classes are correctly classified. Because the patient's symptoms fall under this label, they have no relationship with other classes.

## Conclusion and future work

We have demonstrated the Afaan Oromo intelligent based model, where symptoms of the patient are provided, and the model would categorize disease under a specific group. In this work, we collected the histories of 4500 patients and prepared three separate datasets, AOPS1, AOPS2, and AOPS3. Each document in the dataset is grouped under ten predefined class names with the guidance of a domain expert. This raw data is not directly suitable for training the model, so we applied different preprocessing techniques to clean it and make it important for machines to recognize this sequence of text. Since pre-trained word2vec is not well-suited to Afaan Oromo, the researchers develop word2vec using current datasets. After preprocessing, we have experimented with both ML and DL methods in our study. The performance comparison of all the selected algorithms is done. The main contribution of our work lies in the use of LSTM approaches with trained word-to-vector embedding. The models have also been experimentally analyzed against other state-of-the-art approaches. We concluded our investigations on an AOPS3 dataset since it contains more patient symptoms and found that LSTM techniques produce superior results than other models with 95.7% accuracy and 96.0% F1 score.

These experimental results justify the effectiveness of the proposed methodology in disease category prediction for Afaan Oromo. Although the results show promise, the datasets being utilized are not as large as those employed in the big data cultures that are widespread in the modern era. Thus, the researcher’s future path will be to test the proposed methodology on large, high-quality datasets to evaluate the effectiveness of the prediction model and examine the performance of ensemble classifiers and meta-learning. Additionally, the future methodology must also include other types of health data domains to address issues that arise during data processing.

## Data Availability

The datasets used during the current study will be available from the corresponding author upon request.
